# Membrane Fusion Involved in Neurotransmission: Glimpse from Electron Microscope and Molecular Simulation

**DOI:** 10.3389/fnmol.2017.00168

**Published:** 2017-06-07

**Authors:** Zhiwei Yang, Lu Gou, Shuyu Chen, Na Li, Shengli Zhang, Lei Zhang

**Affiliations:** ^1^Department of Applied Physics, Xi’an Jiaotong UniversityXi’an, China; ^2^Department of Applied Chemistry, Xi’an Jiaotong UniversityXi’an, China; ^3^School of Life Science and Technology, Xi’an Jiaotong UniversityXi’an, China

**Keywords:** membrane fusion, neurotransmission, neurotransmitter release machinery, electron microscope, molecular simulation

## Abstract

Membrane fusion is one of the most fundamental physiological processes in eukaryotes for triggering the fusion of lipid and content, as well as the neurotransmission. However, the architecture features of neurotransmitter release machinery and interdependent mechanism of synaptic membrane fusion have not been extensively studied. This review article expounds the neuronal membrane fusion processes, discusses the fundamental steps in all fusion reactions (membrane aggregation, membrane association, lipid rearrangement and lipid and content mixing) and the probable mechanism coupling to the delivery of neurotransmitters. Subsequently, this work summarizes the research on the fusion process in synaptic transmission, using electron microscopy (EM) and molecular simulation approaches. Finally, we propose the future outlook for more exciting applications of membrane fusion involved in synaptic transmission, with the aid of stochastic optical reconstruction microscopy (STORM), cryo-EM (cryo-EM), and molecular simulations.

## Introduction

**Figure 1:**
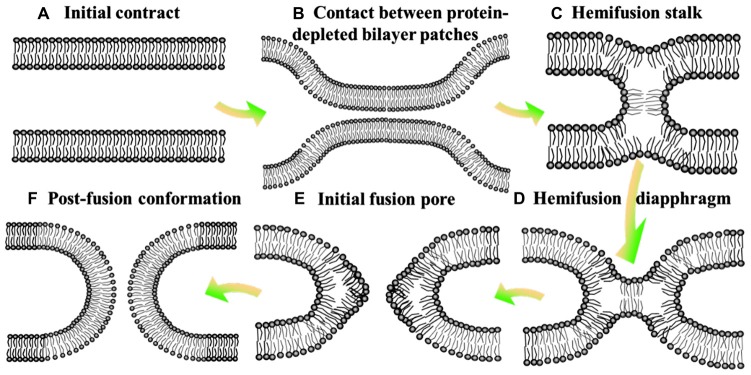
Hemifusion intermediate during the process of membrane fusion. At the state of initial contact **(A)**, lipid bilayers move apart to allow local close contact between two membrane bilayers which proteins mediate membrane binding and fusion **(B)** and a merger of their contacting leaflets into a stalk like hemifusion connection **(C)** that expands into a small hemifusion diaphragm **(D)**. An initial fusion pore opens in a HD **(E)**. This pore gives rise to an hourglass fusion pore **(F)**, expansion of which completes the fusion reaction. There show the bilayer surfaces formed by lipid polar heads.

Neurotransmission is composed of the delivery of neurotransmitters from presynaptic neuron to another neuron, and the feedback of postsynaptic neuron (Jahn and Scheller, 2006; Burnstock, 2007). It is a chemical event which is involved in the transmission of the impulse (Xu et al., 2017), and relies on: the availability of the neurotransmitter; the neurotransmitter release (exocytosis); the binding of the neurotransmitter to the postsynaptic receptor, the excitatory-inhibitory interaction in the postsynaptic cell (Bonifacino and Glick, 2004); and the subsequent removing or deactivating of the neurotransmitter (Iversen, 1971; Heuser and Reese, 1973). Hence, this process requires the controlled release of neurotransmitter from synaptic vesicles by membrane fusion with the presynaptic plasma membrane (Martens and Mcmahon, 2008). Soluble N-ethylmaleimidesensitive factor attachment protein receptors (SNAREs) are the core constituents of the protein machinery which is responsible for synaptic membrane fusion (Jahn and Scheller, 2006). In general, the SNAREs-mediated fusion event is thought to involve a hemifusion diaphragm between the fusion talk and the fusion pore (hemifusion intermediate, Figure 1), where the outer lipid bilayers have been fused, whereas not the inner ones (Zimmerberg, 1987; Brunger et al., 2015). Beyond that, there exists a direct pathway where pre-fusion contact translates into fusion pore without the hemifusion state (Gerst, 1999; Xu et al., 2005; Brunger et al., 2015).

An integral part in the SNAREs-mediated fusion is the assembly of synaptic vesicle transmembrane protein (synaptobrevin) with the target plasma membrane proteins synaptosome-associated protein with relative molecular mass 25 K (SnAP25) and syntaxin (Bommert et al., 1993), which is thought to provide the driving force for the fusion (Haucke et al., 2011). During this process, the extended α-helices of these proteins trend to assemble together, with the formation of four-helix bundles (Mehta et al., 1996). The formed trans-SNARE complex then facilitates the close proximity of vesicular and plasma membranes (~3–4 nm), and induces the membrane fusion (Martens and Mcmahon, 2008).

The spatial regulation of membrane shape, curvature and fluidity are strongly influenced by the lipid composition and topology, during the processes of membrane fusion and fission. Effective neurotransmission requires the precise spatial regulation of lipid-protein interactions for synaptic vesicle targeting, docking, priming and fusion at the active zone (Rohrbough and Broadie, 2005). For an in-depth understanding of the neuronal membrane fusion, various points and states will be summarized in this article, including the process and mechanism of membrane fusion, electron microscope (EM) approaches and molecular simulation results.

## Process and Mechanism of Membrane Fusion

Membrane fusion is identified as a process where two separate phospholipid bilayers merge into an interconnected structure. It is a fundamental physiological and pathological process at the level of cell, organelle and vesicle, resulting in the mixing of the two bilayers of lipids and proteins, as well as the mixing of the contents (Jahn et al., 2003).

### Process of Membrane Fusion

Despite derived by diverse proteins, all fusion reactions processes four fundamental steps (Jahn and Südhof, 1999): membrane aggregation (approaching each other), membrane association (coming into a very close apposition), lipid rearrangement (highly-localized lipid rearrangements of adjacent two bilayers) and lipid and content mixing (complete fusion; Figure 1; Wilschut and Hoekstra, 1986; Plattner et al., 1992; Blijleven et al., 2016).

Hemifusion of lipid bilayers is an important intermediate state in the membrane fusion, which might be boosted by negative spontaneous curvature of monolayer (monolayer trends to bulge toward the hydrophobic tails) and deformation of monolayer induced by the distortion of lipid monolayer (inclusion of amphiphilic peptides; Figure 1; Chernomordik et al., 2006). In addition, bilayers hemifuse when brought to distances of the polar heads of lipids much smaller than the one in the equilibrium state, by adding polyethylene glycol to draw water from the contact zone or by a direct dehydration of multileveled lipid sample. To complete the fusion process, the hemifusion state should proceed to a full fusion pore (Geisow and Fisher, 1986). The pore might open directly from a fusion stalk (stalk-pore pathway) or from a hemifusion state with discernible hemifusion intermediates (hemifusion-fusion pathway; Chernomordik et al., 2006). The formation and closure of the fusion pore are usually regulated by the conformational change with a high activation energy and phase separated lipids, respectively (Oberhauser et al., 1992). The hemifusion diaphragm is a possible intermediate between the stalk and the final fusion pore (Chernomordik et al., 2006). However, Diao et al. (2012) observed more fast fusion (on the ms scale) upon Ca^2+^ addition starting from a hemifusion-free state. This discovery revealed that the neurotransmitter release (especially the fastest event) is more dependent on the immediate pathway, and then stimulated a substantially revised membrane fusion paradigm for the membrane fusion (Wickner and Rizo, 2017).

### Fusion of Protein-Free Lipid Bilayers

The major constituent of the most bilayer lipid, especially the membranes of mammalian cells, is phosphatidylcholine (PC) which has no spontaneous fusion for hours or days (Wang, 2010). Only the tension (or dehydration contact zones) of these bilayers could fuse by the interposition of polyethylene glycol (Sharma and Lindau, 2016). A monolayer protruding into the layer of polar heads seems to consist of molecules with a reasonable inverted cone (Epand, 1998) and positive spontaneous curvature (Chernomordik et al., 2006). A lipid monolayer that orients toward the hydrocarbon tails usually possesses a negative curvature, composed by cone shaped lipid molecules (Chizmadzhev, 2004). A possible explain is that the action of proteins in fusion process do not directly facilitate the formation of fusion intermediates, also generate fusogenic lipids (sphingomyelinase and phospholipase; Epand, 2000; Chernomordik and Kozlov, 2008). It is promoted by the defects created of the bilayers referring membrane perturbation, including the vicinity of lipid phase transition, the separation of lateral phase or the generation of domain, the high local curvature of membrane, osmotic or electric stress in or on the membrane; the present of amphipaths or macromolecules within the membrane, etc. (Cevc and Richardsen, 1999). High concentrations of lysolipids, which increase the intrinsic curvature of the monolayer, inhibit several biological membrane fusion processes (Shangguan et al., 1996; Söllner, 2004).

### Protein-Mediated Membrane Fusion

The context of membrane fusion *in vivo* is more complicated since biological fusion is always mediated by protein. However, the specific mechanisms of these processes still remain elusive, especially on proteins which promote the development of hemifusion and fusion pore. Viral membrane fusion and synaptic membrane fusion are widely studied among current protein-mediated membrane fusions. The former promotes the combine between the viral membrane and the host cell membrane, then inducing the release of viral genome into the cytoplasm, as well as the replication cycle of virus. There are two mechanisms of activating viral fusion proteins: exposure to low pH and pH-independent (Earp et al., 2005).

The latter carries the neurotransmitter across the synapses (neurotransmitter release) and plays an important role in the signals traveling in the central nervous system. Neurotransmitter release during the synaptic membrane fusion process requires a protein family that have termed SNAREs which can be divided into four categories (Diao et al., 2012; Hughson, 2013): (1) vesicle-anchored (v) and target-membrane–anchored (t) SNAREs; (2) N-ethylmaleimide–sensitive factor (NSF) and NSF attachment proteins (SNAPs); (3) Rab GTPases and multicomponent vesicle tethering complexes; and (4) Sec1/Munc18 (SM) proteins. So far, the mechanism of this family still has a few basic doubts, such as function, conformational changes, etc. The SNARE-mediated membrane fusion was conducted the zippering mechanism which pulls two membranes together (Diao et al., 2013b). In general, synaptic membrane fusion requires a consecutive two-step pathway. First, the N-terminal domain of the vesicle (v-) SNARE, synaptobrevin-2, docks to the target membrane (t-) SNARE, thereby results in a conformational rearrangement of a half-zippered SNARE complex. Then, the assembled SNARE complex locks the C-terminal portion of the t-SNARE into the same way as the four-helix bundle, which is formed with syntaxin and SNAP-25. Besides, this fusion is greatly accelerated by synaptotagmin-Ca^2+^ (Lai et al., 2013). Reconstitutions of synaptic vesicle fusion indicated that the interactions between the Ca^2+^-binding loops of the synaptotagmin-1 and phospholipids are critical to release of neurotransmission, when little content mixing occurs in the absence of Ca^2+^ (Diao et al., 2013b; Wickner and Rizo, 2017).

## Electron Microscope on Membrane Fusion

In general, neuronal communication is mediated by neurotransmitters release induced by Ca^2+^-induced synaptic vesicle exocytosis. It is a long-sought goal that understanding the mechanism of synaptic vesicle fusion, associated with the development of *in vivo* synthetic system. With the advent of modern electron microscopic techniques, we could particularly investigate the neurotransmission and the consequent membrane alterations.

**Figure 2:**
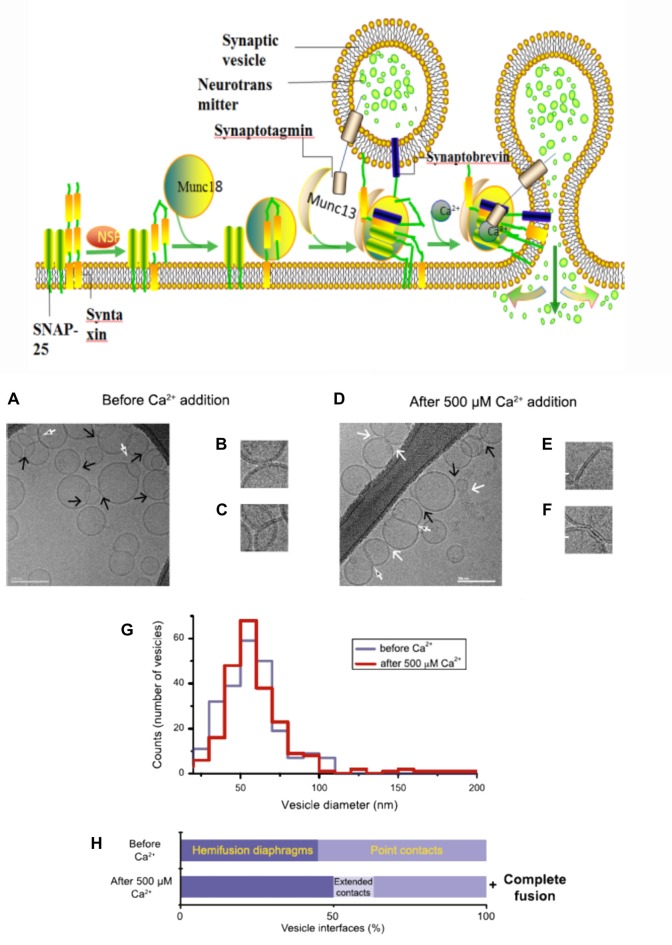
A model with Muncs (Upside) the Soluble N-ethylmaleimidesensitive factor attachment protein receptor (SNARE) complex, fused by v-liposomes (containing synaptobrevin) and t-liposomes (containing SNAP-25 and syntaxin), was disassembly derived under the N-ethylmaleimide–sensitive factor (NSF) with the consuming of adenosine triphosphate (ATP). The entry of Munc18-bound syntaxin into SNARE complexes was catalyzed by Munc13, with the assembly of ternary v/t-SNARE complexes. Then Synaptotagmin-Ca^2+^ induce the release of neurotransmitter, thereby drive membrane fusion. Imaging of donor/acceptor interface morphologies by cryo-electron microscopy (EM) before and after Ca^2+^ addition (Downside; Diao et al., 2012).

During neurotransmitter release, several SNARE protein complexes involve with synaptobrevin (vesicle (v-) SNAREs) and syntaxin and SNAP-25 (target membrane (t-) SNAREs) to mediate the fusion of two membranes. Meanwhile, this vesicle membrane fusion is acutely triggered in a Ca^2+^-dependent manner (Figure 2). Munc18 (also called neuronal Sec1) forms a tight complex with syntaxin, with a closed conformation that is unable to bind other SNAREs. Regarding as Munc13, a synaptic vesicle “priming” protein, catalyzes the transition from syntaxin-Munc18 complex to fully assembled v/t-SNARE complex (syntaxin—SNAP-25—synaptobrevin), via bridging the vesicle and plasma membranes and controlling vesicle tethering (Hughson, 2013; Ma et al., 2013; Wickner and Rizo, 2017). Chen et al. (2002) explored the atomic structure of the complexin/SNARE complex, using the X-ray and TROSY-based NMR methods. The results revealed that complexin presents an antiparallel helical conformation, stabilizes the interface between two helices of synaptobrevin and syntaxin, thus enables the Ca^2+^-evoked neurotransmitter release with the exquisitely high speed.

Over the past 10 years, cryo-electron microscopy (cryo-EM) has been developing rapidly, which combines the potential of three-dimensional (3D) imaging at molecular resolution with a close-to-life preservation of biological samples. Rapid freezing followed by the investigation of the frozen-hydrated samples avoids the artifacts caused by chemical fixation and dehydration procedures. Furthermore, the biological material is observed directly, without heavy metal staining, avoiding artifacts caused by the unpredictable accumulation of staining material (Lucić et al., 2005). The vesicle clusters induced by Ca^2+^-bound C_2_ domains of synaptptagmin-1 was visualized by the Cryo-EM method, and the tomographic 3D reconstruction of a vesicle cluster revealed that this process might be induced by Ca^2+^-dependent phospholipid binding of the C_2_AB fragment, where the C_2_B domain cooperates with the SNAREs bring the membranes together, as well as the multiple interactions between the C_2_B domain and phospholipids (diacyglycerol and phosphatidylinositol 4,5-bisphosphate (PIP_2_); Araç et al., 2006).

In fact, there is still an active debate regarding whether SNAREs are linked to pre-fusion contact to a fusion pore or participate later in the fusion process by facilitating hemifusion, through the formation of tight SNARE complexes and gathering of the vesicle and plasma membranes. With the aid of single-vesicle fluorescence fusion assay and EPR, the direct observation of two-faceted functions of complexin revealed the formation of a complex substrate (SNARE complexes, complexins and phospholipids) for Ca^2+^ and Ca^2+^-sensing fusion effectors in the release process of neurotransmitter (Yoon et al., 2008). Neuron firing gives rising of the intracellular Ca^2+^ concentration, with the triggering of synaptic vesicles fusion to carry neurotransmitter molecules. Diao et al. (2012) used recently developed Cyro-EM method to monitor the temporal sequence of both content and lipid exchange upon Ca^2+^-triggering between single pairs of donor and acceptor vesicles on a 100-ms time scale (Diao et al., 2012). Their system performed a quantitative analysis of all observed cryo-EM images (before and after Ca^2+^-injection) and achieved a Ca^2+^ sensitivity in the 250–500 μM range (Figure 2). During their experiments, hemifusion diaphragms were observed, as well as points where liposomes contacted each other without the shape change of membrane. Extended tight contacts of membrane were not observed, without the present of Ca^2+^. With the addition of Ca^2+^, there merely exists the process from point-contacts to fast fusion (Diao et al., 2012). It was found that alone neuronal SNAREs cannot efficiently induce the complete fusion. The combination of SNAREs with selected components (small-head group lipids, Munc18-1, Munc 13 and synaptotagmin-Ca^2+^) could lower the activation barriers during the fusion process, because of enhancing the kinetic control by complexin (Kyoung et al., 2011; Wickner and Rizo, 2017). Bharat et al. (2014) performed reconstitutes synaptic fusion and applied large-scale, automated cryo-electron tomography to observe this *in vitro* system. Afterwards docking and priming of vesicles with the fast Ca^2+^-triggered fusion, a local protrusion in the plasma membrane will be induced by the SNARE proteins, with the direction towards the primed vesicle and allowing synchronous and instantaneous fusion upon the complexin clamp release (Zhang et al., 2015b).

## Molecular Simulation on Membrane Fusion

Many efforts have been devoted to modeling the membrane fusion process involved in synaptic transmission via molecular simulations, such as Coarse-grained (CG) molecular dynamics (MD) simulations. The results of these studies revealed the mechanics of membrane fusion involved in synaptic transmission and some key physical properties of lipid monolayers and related proteins.

The SNARE complex between opposing membranes promotes membrane fusion of synaptic transmission (Mayer, 1999; Pfeffer, 1999). *In vivo*, the formation of complex connects the opposing membranes and pulls two membranes together using their α-helical transmembrane domains (TMD; Ossig et al., 2000). In addition, SNARE complexes are also deemed to overcome the fusion barriers and to accelerate the fusion process (Chen and Scheller, 2001; Hong, 2005; Risselada and Grubmüeller, 2012).

The SNARE complex is represented by a twisted bundle of four α-helices which generally consists of SNAP-25, syntaxin-1, and synaptobrevin-2, confirmed by coarse-grain MD simulations (Durrieu et al., 2009; Tekpinar and Zheng, 2014) There is mechanistically link between the conformational flexibility of SNARE TMD helices and their ability to induce lipid mixing (Nagy et al., 2005; Stelzer et al., 2008). The basic residues (positive charged) at the C terminal of SNAP-25 is benefit for the tight zippering of SNARE complex and the binding with negatively charged lipid head groups, improving the high frequency and clipping neurotransmitter release (Fang et al., 2015). Besides, the transmembrane domain of synaptobrevin II (sybII TMD) influences both the natural helicity and flexibility of SNARE (Gao et al., 2012; Zheng, 2014; Han et al., 2016). The assemblage of SNARE complex is regulated by complexin, a cytoplasmic neuronal protein and the MD results suggest that the α-accessory helix of complexin (Cpx AH) make partially unzipped state of the SNARE bundle being stabilized by its functions in relate in the clamping of synaptic vesicle fusion (Ghahremanpour et al., 2010; Bykhovskaia et al., 2013; Lai et al., 2014, 2016; Gong et al., 2016).

**Figure 3:**
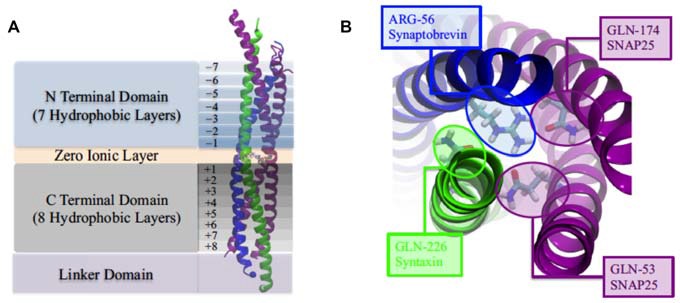
**(A)** The four domains of neuronal SNARE protein: N Terminal Domian, Zero Ionic Layer, C Terminal Domain, Linker Domain; and involved four helices: snaptobrevin (blue), syntaxin (green), synaptosome-associated protein with relative molecular mass 25 K (SNAP25; magenta). **(B)** Top view of zero ionic layer. Reproduced with permission from Tekpinar and Zheng (2014).

**Figure 4:**
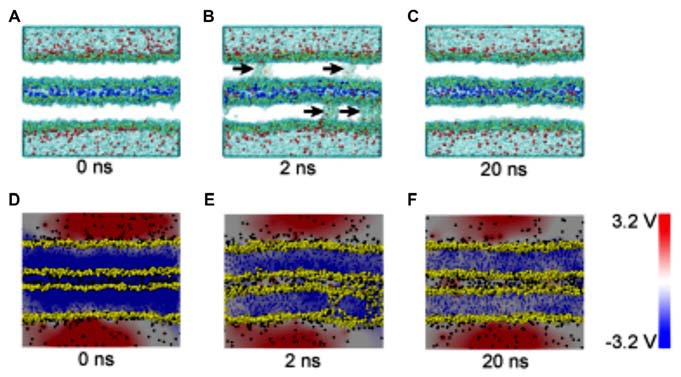
Snapshots and electrical potential distributions during the process of fusion pore (membrane contact-pore formation-membrane healing) through the molecular dynamics (MD) simulations **(A–C)**: the process of fusion pore formation. **(D–F)**: electric potential alteration during the process (Bu et al., 2016).

The composition of several parts of the SNARE completes its function of modulate membrane fusion (Figure 3). MD simulations have also been used in the study of other functions in relation with membrane fusion. During the synaptic transmission, vesicles filled with neurotransmitter molecules are required to be docked to the membrane (Knecht and Grubmüller, 2003; Bock et al., 2010; Lai et al., 2015). Therefore, the SNARE complex use attractive forces to counterbalance the long-range repulsion between the vesicle and membrane (Diao et al., 2013a; Fortoul et al., 2015). More recently, the formation of a transient pore by using the MD method was first reported. The close contact of two membranes gives rise to a high local transmembrane voltage. The decrease of the distance of the opposed bilayers brings out the increase of the transmembrane voltage. When the distance is under a critical value, the local transmembrane voltage is enough high to induce the transient of membrane pores (Figure 4; Ribrault et al., 2011; Bu et al., 2016). Finally, some findings have offered new structural and dynamic details of SNARE disassembly mechanism based on CG modeling (Zheng, 2016).

## Outlook

The controlled release of neurotransmitter by membrane fusion, from synaptic vesicles to presynaptic cell, is an important step in the synaptic transmission (Trimbuch and Rosenmund, 2016). This universal fusion can be accelerated by synaptotagmin-Ca^2+^, with the assembly of specialized proteins (such as SNAREs) within the opposing membrane bilayers. Electron density map, 3-D topography and simulation studies of the SNARE ring complex, advance that membrane-associated SNAREs overcome repulsive forces to process the two membranes being close to each other (just 2.8 Å apart; Chen and Scheller, 2001). However, people are actually working on an assumption that all proteins are at the right position for inducing membrane fusion. Since all proteins are unlabeled, one is not able to tell the real story on the protein side, which is also an important part for the study of membrane fusion in synaptic transmission. Thus, in the future, it requires the high spatial resolution techniques in order to monitor these interactions simultaneously during the fusion processes, such as stochastic optical reconstruction microscopy (STORM), is required (Diao et al., 2011). In STORM, single biomolecules containing photo-switchable fluorophores are turned on and off repeatedly, to find their positions precisely with ~20 nm resolution by determining the center position of the point-spread function from reconstructed images for each biomolecule (Rust et al., 2006).

Cryo-electron microscopy, abbreviated as “cryo-EM”, is a form of transmission EM (TEM) technique which observes the sample (generally biological sample) at cryogenic temperatures in order to void the ultrastructural changes (Doerr, 2016). It has been rapidly developed in the decade, with increasing popularity in structural biology (Callaway, 2015; Nogales et al., 2016). As cryo-EM became matured, it has been adopted by an ever-increasing range of disciplines to offer tools for providing a cell-like yet simplified environment for investigating the membrane fusion, especially the dynamic structural change of important proteins and the dynamic mechanism of fusion process. In particular, optimized negative-staining (OpNS) EM images have revealed several important physical attributes of CETP and substantial molecular basis for the CETP-mediated lipid exchange (Zhang et al., 2012, 2015a).

Active zones of synaptic plasma membranes are known to concentrate the components that drive membrane fusion, such as the SNAREs, Munc 18-1, Munc 13-1 and small-head group lipids (e.g., diacyglycerol and PIP2), while the participation and characteristic of these macromolecular complexes are still not fully understood (Rizo and Xu, 2015; Ryu et al., 2016; Wickner and Rizo, 2017). Strikingly, current experimental techniques do not achieve a resolution better than ms/μs in time, and neuronal membrane fusion normally occurs at ms timescale (Diao et al., 2012). CG MD simulation is one of effective solutions to overcome the time-scale gap between computational and experimental methods (Bhushan, 2016). With the aid of residue-based and shape-based CG approaches, the regulatory mechanisms of SNARE proteins have been briefly outlined, focused on the intricate molecular mechanisms between proteins and membranes (Bu et al., 2016; Zhang et al., 2016; Han et al., 2017). However, the application of CG models sacrifice degrees of freedom and accurate molecular interactions to get the requirement of less resources (Bhushan, 2016). Though the prohibitive computational cost usually limits the simulation times and system sizes of all-atom models less than 1000 ns and 10 nm, it will provide the description of SNARE-mediated membrane fusions with all-atom detail, such as the specific lipid properties for stimulating fusion, the tethering/SM protein complex, the lipid-protein (such as Munc18-1, Munc13-1, and complexin) interactions, and membrane architecture. Nevertheless, molecular simulation selectivity leads to the factitious results of synaptic membrane fusion, therefore, the computational methods and initial models should be amending continuously by the sufficient basic parameters derived from the non-invasive experimental observations (such as STORM and cryo-EM; Diao et al., 2013b; Wickner and Rizo, 2017).

As STORM, cryo-EM, and all-atoms molecular simulations continue to develop through advances in technological innovation, the combination of the three techniques will be a powerful tool for the in-depth investigation on the regulatory mechanisms of synaptic membrane fusion at atomistic resolution, uncovering the recruitment process of Sec1-Munc 18 family proteins to catalyze SNARE assembly, specific lipid properties which be crucial for fusion, and the intricate balance of protein-lipid interactions. We expect to see more exciting applications of synaptic membrane fusion with continued advances in these methods.

## Author Contributions

The manuscript was initially drafted by ZY, SZ and LZ and then further edited after discussion with LG, SC and NL.

## Conflict of Interest Statement

The authors declare that the research was conducted in the absence of any commercial or financial relationships that could be construed as a potential conflict of interest. The reviewer RS and handling Editor declared their shared affiliation, and the handling Editor states that the process nevertheless met the standards of a fair and objective review.
